# RETRACTION: Analysing Predictors of Surgical Site Infections in Patients Undergoing Emergency Surgery for Traumatic Pulmonary Haemorrhage

**DOI:** 10.1111/iwj.70501

**Published:** 2025-04-02

## Abstract

**RETRACTION:** ZhangJ. , PanC. , “Analysing Predictors of Surgical Site Infections in Patients Undergoing Emergency Surgery for Traumatic Pulmonary Haemorrhage,” International Wound Journal 21, no. 4 (2024): e14860, 10.1111/iwj.14860.38572791 PMC10993333

The above article, published online on 04 April 2024, in Wiley Online Library (http://onlinelibrary.wiley.com/), has been retracted by agreement between the journal Editor in Chief, Professor Keith Harding; and John Wiley & Sons Ltd. Following an investigation by the publisher, all parties have concluded that this article was accepted solely on the basis of a compromised peer review process. The editors have therefore decided to retract the article. The authors did not respond to our notice regarding the retraction.

**RETRACTION:** J. Zhang , C. Pan , “Analysing Predictors of Surgical Site Infections in Patients Undergoing Emergency Surgery for Traumatic Pulmonary Haemorrhage,” International Wound Journal 21, no. 4 (2024): e14860, 10.1111/iwj.14860.38572791 PMC10993333

The above article, published online on 04 April 2024, in Wiley Online Library (http://onlinelibrary.wiley.com/), has been retracted by agreement between the journal Editor in Chief, Professor Keith Harding; and John Wiley & Sons Ltd. Following an investigation by the publisher, all parties have concluded that this article was accepted solely on the basis of a compromised peer review process. The editors have therefore decided to retract the article. The authors did not respond to our notice regarding the retraction.

